# The Effectiveness and Cost-Effectiveness of Screening for HIV in Migrants in the EU/EEA: A Systematic Review

**DOI:** 10.3390/ijerph15081700

**Published:** 2018-08-09

**Authors:** Kevin Pottie, Tamara Lotfi, Lama Kilzar, Pamela Howeiss, Nesrine Rizk, Elie A. Akl, Sonia Dias, Beverly-Ann Biggs, Robin Christensen, Prinon Rahman, Olivia Magwood, Anh Tran, Nick Rowbotham, Anastasia Pharris, Teymur Noori, Manish Pareek, Rachael Morton

**Affiliations:** 1 Bruyère Research Institute, 85 Primrose Ave, Annex E, Ottawa, ON K1R 7G5, Canada; prinon.rahman@dal.ca (P.R.); omagwood@bruyere.org (O.M.); 2 Departments of Family Medicine & Epidemiology and Community Medicine, University of Ottawa, Ottawa, ON K1N 6N5, Canada; 3 Faculty of Health Sciences, American University of Beirut, Beirut 1107 2020, Lebanon; tamara_loutfi@hotmail.com (T.L.); lmk22@mail.aub.edu (L.K.); ea32@aub.edu.lb (E.A.A.); 4 AUB GRADE Center, Clinical Research Institute, American University of Beirut, Beirut 1107 2020, Lebanon; 5 Department of Internal Medicine, American University of Beirut, Beirut 1107 2020, Lebanon; paa15@mail.aub.edu (P.H.); nr00@aub.edu.lb (N.R.); 6 National School of Public Health, Centro de Investigação em Saúde Pública & GHTM/IHMT, Universidade Nova de Lisboa, 2825-149 Caparica, Portugal; smfdias@yahoo.com; 7 Department of Medicine/RMH at the Doherty Institute, The University of Melbourne Vic Australia, Parkville 3010, Australia; babiggs@unimelb.edu.au; 8 Musculoskeletal Statistics Unit, The Parker Institute, Bispebjerg and Frederiksberg Hospital, 2000 Frederiksberg, Denmark; Robin.Christensen@regionh.dk; 9 Department of Rheumatology, Odense University Hospital, 5000 Odense, Denmark; 10 NHMRC Clinical Trials Centre, The University of Sydney, Campbell 2006, Australia; anh.tran@ctc.usyd.edu.au (A.T.); rowbothamn@gmail.com (N.R.); Rachael.morton@ctc.usyd.edu.au (R.M.); 11 European Centre for Disease Prevention and Control, 16973 Solna, Sweden; Anastasia.pharris@ecdc.europa.eu (A.P.); teymur.noori@ecdc.europa.eu (T.N.); 12 Department of Infection, Immunity and Inflammation, University of Leicester, Leicester LE1 7RH, UK; mp426@le.ac.uk

**Keywords:** HIV, AIDS, stigma, refugees, migrants

## Abstract

Migrants, defined as individuals who move from their country of origin to another, account for 40% of newly-diagnosed cases of human immunodeficiency virus (HIV) in the European Union/European Economic Area (EU/EEA). Populations at high risk for HIV include migrants, from countries or living in neighbourhoods where HIV is prevalent, and those participating in high risk behaviour. These migrants are at risk of low CD4 counts at diagnosis, increased morbidity, mortality, and onward transmission. The aim of this systematic review is to evaluate the effectiveness and cost-effectiveness of HIV testing strategies in migrant populations and to estimate their effect on testing uptake, mortality, and resource requirements. Following a systematic overview, we included four systematic reviews on the effectiveness of strategies in non-migrant populations and inferred their effect on migrant populations, as well as eight individual studies on cost-effectiveness/resource requirements. We assessed the certainty of our results using the Grading of Recommendations Assessment, Development, and Evaluation (GRADE) approach. The systematic reviews reported that HIV tests are highly accurate (rapid test >90% sensitivity, Western blot and ELISA >99% sensitivity). A meta-analysis showed that rapid testing approaches improve the access and uptake of testing (risk ratio = 2.95, 95% CI: 1.69 to 5.16), and were associated with a lower incidence of HIV in the middle-aged women subgroup among marginalised populations at a high risk of HIV exposure and HIV related stigma. Economic evidence on rapid counselling and testing identified strategic advantages with rapid tests. In conclusion, community-based rapid testing programmes may have the potential to improve uptake of HIV testing among migrant populations across a range of EU/EEA settings.

## 1. Introduction

Migrants, encompassing a broad range of subgroups (i.e., asylum seekers, refugees, undocumented and economic migrants, foreign students, etc.), are individuals who move from their country of residence to another. Populations at unique high risk for human immunodeficiency virus (HIV) include migrants from countries where HIV is prevalent, living in neighborhoods where HIV is prevalent, and participating in high-risk behaviour [1]. In 2016, 29,444 new HIV infections were diagnosed in the European Union/European Economic Area (EU/EEA) [2]. Although the overall number of HIV diagnoses in migrants from high-prevalence countries have declined in the EU/EEA over the past decade, migrants still accounted for 40% of the reported cases in 2016 (range 1–80%) [2]. HIV acquisition among migrants was thought to occur pre-migration, but recent EU/EEA evidence [3,4,5,6] suggests post-migration acquisition is also of concern and this suggests community based targeted interventions, including screening programmes, which may be needed many years after arrival to the EU/EEA. About 15% of all people living with HIV (*n* = 122,000) in the EU/EEA are unaware of their HIV positive status [7], making accurate data on HIV prevalence among migrant populations in the EU/EEA difficult. Those with an increased risk of HIV infection include migrants from HIV endemic regions, men who have sex with men (MSM), those with multiple sex partners, and injection drug users (IDU) [8].

In an effort to scale up diagnosis and treatment programmes, the Joint United Nations Programme on HIV/acquired immunodeficiency syndrome (AIDS) (UNAIDS) outlined the 90–90–90 treatment target to help end the AIDS epidemic. By 2020, 90% of those with HIV will know their status, 90% of all people with HIV will receive sustained antiretroviral therapy (ART), and 90% of all people receiving ART will have viral suppression [9]. In response to the variability in the testing and treatment approaches for HIV across EU/EEA countries, the European Centre for Disease Prevention and Control (ECDC) published guidelines on HIV testing [10]. Most countries have reported having guidance for HIV testing at the ational level [11,12]. At least 22 countries acknowledge that migrants are vulnerable to HIV infection, but six of these do not explicitly recommend HIV testing options for migrant populations [13]. Additionally, these testing options are often inconsistent between and within countries. Currently, there are no EU/EEA-wide HIV testing recommendations or strategies specifically tailored for migrant populations.

Conventional HIV testing is defined as traditional laboratory testing in healthcare settings, where patients have to wait days to weeks to receive their results; this testing approach includes an ELISA test followed by a Western blot. Rapid HIV testing refers to voluntary enrolment where results are obtained within hours, followed by a confirmatory test, and with links to outreach counselling for results and treatment options [14]. Rapid testing strategies are feasible for field settings, and the WHO recommends rapid testing as part of the community based testing strategy for communities with persistent HIV-related stigma [15].

In 2015, the World Health Organization (WHO) published consolidated guidelines on the use of antiretroviral drugs for treating and preventing HIV infection [15]. This public health approach advocated by the WHO considers the collective health status of a population, to ensure wide access to high quality services using simplified and standard HIV testing approaches, such as conventional, community-based, and other rapid testing techniques [15]. Migrants from Sub-Saharan Africa and Latin America are more likely to be diagnosed late (defined as having a CD4 count of less than 350 CD4+ cells/mm^3^) in comparison to non-migrant Europeans [2,8]. Late diagnosis increases the disease transmission rate, and increases the risk of morbidity and mortality [16]. The main reasons for late diagnosis among migrants are believed to include impaired access to testing as a result of HIV-related stigma, fear, guilt, economic difficulties, and difficulties accessing health care in Europe [8]. This review focuses on the newly arrived migrants to the EU/EEA, who migrated within the past five years, with consideration given to country and origin, circumstances of migration, gender, and age, where relevant. This group of migrants is often less well integrated into health systems because of a lack of reliable access to health services, poor information about healthcare, lack of supportive language provision, and inattention to the gender dimensions of healthcare [17]. While marginalized migrants were the specific focus, we recognize that other migrant populations may also benefit from this review. We conducted a systematic review on the effectiveness, as well as a second systematic review on the cost-effectiveness, of screening for HIV among migrants in the EU/EEA region, with the aim of informing the development of ECDC migrant screening guidance.

## 2. Methods

Using the Grading of Recommendations Assessment, Development, and Evaluation (GRADE) approach; the Campbell and Cochrane Collaboration Equity Methods Group; and a review team including clinicians, public health experts, and researchers from across the EU/EEA, to conduct the evidence syntheses. A detailed description of the methods can be found in the registered systematic review protocol [18].

The review group followed the PRISMA reporting guidelines [19] for the reporting of this systematic review (PROSPERO [CRD42016045798]). In summary, the review team developed key research questions (PICO: population, intervention, comparison, and outcome) and a logic model showing an evidence chain to identify key concepts, to consider potential role of indirect evidence related to populations and interventions, and to support the formulation of search strategies (see Appendix A and Appendix B) [18]. The review teams aimed to answer the following overarching questions.

- Should newly arrived migrants be screened for HIV? Who should be targeted and how?
- What implementation considerations should be considered when screening for HIV in newly arrived migrants to the EU/EEA?

‘Migrants’, a focus for the eligible evidence, included asylum seekers, refugees, undocumented migrants, and other foreign-born residents, with a focus on newly arrived migrants from HIV intermediate (>0.1%) and high (>1%) prevalence countries to EU/EEA in the last five years. Our analysis did not consider specific subgroups of migrants, but rather, it focused on those that were at high risk of exposure and facing poor access to testing and treatment. This review included various rapid testing approaches and provider-initiated testing approaches. Evidence was evaluated using a hierarchical approach, whereby systematic reviews/meta-analyses, and evidence based guidelines were given the most weight, followed by individual randomized controlled trials (RCTs), quasi-experimental studies, and observational studies [20,21]. The availability of existing high quality systematic reviews on these topics led us to follow a review of reviews methodology, thereby excluding all of the articles that were not systematic reviews. The team sought to build on existing high quality evidence and to identify gaps that may exist in the evidence-base.

Relevant search terms and strategies were used to search published literature in Ovid MEDLINE, Database of Abstracts of Reviews of Effects (DARE), Cochrane Database of Systematic Reviews (CDSR), and EMBASE from 2010 to December 2016, and NHS EED, CEA Registry (Tufts University), and Google Scholar from 1995 to 2016 (See Appendix B), and grey literature through Google, as well as the U.S. Centres for Disease Control and Prevention (CDC), ECDC, UNAIDS, and WHO websites. The general search terms used included “HIV”, “AIDS”, “screening”, “early diagnosis”, and “disease surveillance” (see Appendix B for complete search strategy). No language restrictions were applied to the searches. Migrants and refugees were key populations of interest, but we also considered studies that included marginalised populations with a high prevalence of HIV.

Two independent team members (Tamara Lotfi and Lama Kilzar) manually reviewed the titles, abstracts, and full text of identified citations; selected evidence for inclusion; and compiled evidence reviews and PRISMA flow sheets. Disagreements were resolved by consensus or the involvement of a third reviewer. We assessed the methodological quality of the potentially included studies with AMSTAR [22] or Newcastle Ottawa Newcastle Scales [23]. For evidence of cost-effectiveness, we extracted data from relevant study designs (e.g., micro-costing studies, within-trial cost-utility analyses, and Markov models) for three specific questions, namely: the size of the resource requirements, the certainty of evidence around resource requirements, and whether the cost-effectiveness results favoured the intervention or comparator [24]. Finally, we assessed the certainty of the economic evidence in each study using the relevant items from the 1997 Drummond checklist [25]. The team created tables showing the characteristics of the included studies (see Table 1 and Table 2), then rated the certainty of the effects for pre-selected outcome measures, and finally, conducted meta-analyses and created GRADE evidence profiles.

The final analysis report was on the GRADE synthesis. The certainty of the evidence rating reflects the level of confidence in an estimate of the desirable and undesirable effects. The implementation considerations were informed by exisiting literature.

## 3. Results

**Figure 1:**
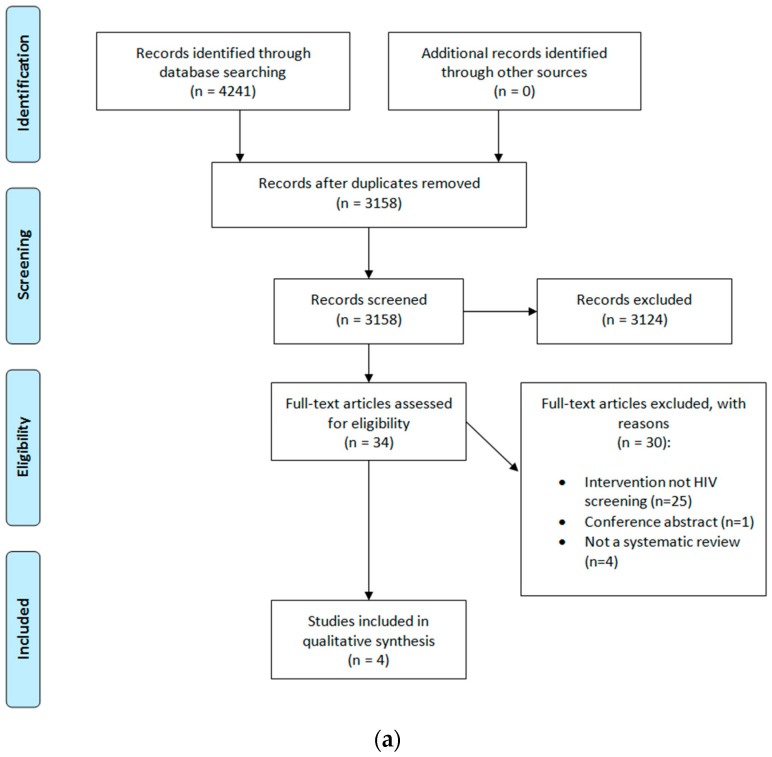

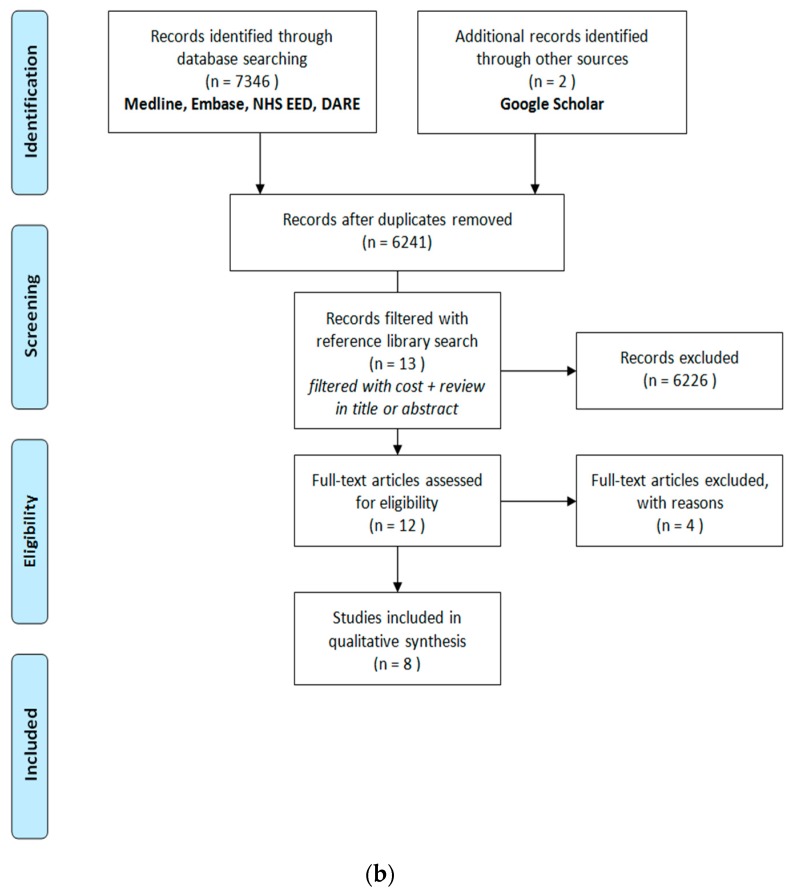
(**a**) Preferred reporting items for systematic review and meta-analysis protocols (PRISMA0 flow diagram (effectiveness); (**b**) PRISMA flow diagram (cost-effectiveness). HIV—human immunodeficiency virus.

We retrieved 4241 articles on the effectiveness of HIV testing options. After the removal of duplicates, 3158 studies were screened by title and abstract for eligibility, based on our PICO criteria (see Table A1 in Appendix C). Of these, 34 studies were screened for full-text, and 30 studies were excluded at the full-text stage. The reasons for exclusion were that the intervention was not HIV testing (*n* = 25), conference abstract (*n* = 1), and not a systematic review (*n* = 4). Four systematic reviews were included in the end [26,27,28,29] (see Figure 1a). Additionally, 7346 economic studies were identified. After the removal of duplicates, we screened 6241 titles and abstracts for eligibility, and filtered the remaining records with “cost” and “review” in the title or abstract. Of the remaining 13 articles, 12 articles were selected for a full-text review. Eight studies were included [30,31,32,33,34,35,36,37] (see Figure 1b). Four studies were excluded, as a result of relevance to our PICO criteria.

Our systematic review evaluated voluntary HIV testing approaches among migrants from HIV intermediate (>0.1%) and high (>1%) prevalence countries arriving to the EU/EEA. This included various rapid testing approaches and provider-initiated testing approaches. Only one randomised-controlled trial (RCT), from the United States [38], explicitly identified migrants within their study population. This study was included in Pottie (2014). The GRADE methodology to assess the certainty of evidence considers differences in the study populations and interventions (indirectness) as a potential reason to downgrade the level of certainty (See Table 3), allowing us to interpret the findings consistently for the migrant populations [39]. None of the systematic reviews contained any RCTs or observational studies comparing clinical outcomes between indiviuals screened or not screened for HIV infection. No RCT or observational study evaluated the value of repeat HIV testing compared with one-time testing, or of different strategies for repeat testing. No studies compared the effects of different pre- or post-test HIV counselling methods on testing uptake or rates of follow up, and linkage to care.

The U.S. Agency for Healthcare Research and Quality (AHRQ) systematic review reported that prior studies have shown that HIV testing was accurate (Rapid Test >90% sensitive, Western blot and ELISA >99% sensitive) [28]. However, the review found that targeted screening programmes, which test patients with identified risk factors, may still have missed a proportion of cases [28]. The universal opt-out rapid testing strategy was associated with a higher likelihood of testing compared with physician-directed, targeted rapid testing (25% vs. 0.8%; relative risk [RR] = 30 [95% CI: 26 to 34]), but not necessarily in marginalised populations [28]. Universal testing was also associated with a higher median CD4 count and lower likelihood of CD4 count <200 cells/mm^3^ at the time of diagnosis, compared with targeted HIV testing, but these differences were not statistically significant [28].

New HIV diagnoses detected through universal testing in the United States had follow-up rates that were reported to be between 75–100% [28]. One study directly compared universal and targeted testing strategies [40]. Both the universal and targeted strategies resulted in very high rates of follow up (defined as attending at least one HIV clinic visit) between 97% and 100% [40]. The sample sizes of the included studies were small (range of 17–74 newly diagnosed HIV infections). The U.S. AHRQ review also reports that the treatment was very effective at improving clinical outcomes in adolescent and adult patients with advanced immunodeficiency [28]. The evidence indicates, from primary studies of included systematic reviews, that treatment reduced the risk of AIDS-defining events and mortality in persons with less advanced immunodeficiency and reduced sexual transmission in discordant couples [41].

In the EU/EEA, migrants from HIV-endemic countries were at a high risk of HIV infection [42]. The groups identified as having a high HIV prevalence were people originating from Sub-Saharan African, Latin America, Southeast Asia, and Eastern Europe [2,42]. These migrants had a higher frequency of delayed HIV diagnosis and are more vulnerable to the negative effects of the disclosure of their HIV status [42]. For migrants from countries where HIV prevalence is low, their socio-economic vulnerability put them at risk of acquiring HIV post-migration [42]. Migrants tended to report high-risk behaviour for HIV, such as multiple sexual partners, low and inconsistent condom use, high alcohol consumption, and drug use [42]. Men who have sex with men (MSM); sex workers, both male and female; and migrant women are considered particularly vulnerable populations within this group [42].

One systematic review and meta-analysis focused on the effectiveness of rapid tests for high-risk populations for HIV exposure. One of the RCTs included migrants-specific [38], and the others involved high-risk marginalised populations. The results of the included systematic review found that rapid voluntary counselling and testing was associated with a large increase in HIV-testing uptake and receipt of results in comparison to conventional testing (RR = 2.95, 95% CI: 1.69–5.16), but these studies did not report on uptake of HIV treatment [26]. The GRADE quality of the included studies was assessed to be low, because of the risk of bias and imprecision. All of the harms of rapid testing were not considered for the scope of the present review.

Repeat testing was found to be more likely among the individuals where rapid testing was performed (RR = 2.28, 95% CI 0.35 to 15.07) [26]. Retesting was also more likely for the individuals who were reminded to re-test by short message service (SMS) text messaging (pooled Odds ratio (OR) 2.19 [95% CI 1.46 to 3.29]) [29]. Receiving phone calls, verbal advice, and/or counselling also resulted in higher rates of retesting than phone calls alone (OR 2.50 [95% CI 1.3 to 4.8]) [29]. In the communities where rapid HIV testing was implemented, the HIV incidence decreased by 11% in comparison to the control arm communities [26]. The evidence for the uptake of HIV testing, receipt of results, and repeat testing were considered of moderate quality, because of randomisation and allocation concerns. In the review that addressed provider initiated treatment and counselling (PITC), nineteen studies were included, all from Sub-Saharan Africa (*n* = 15) or Asia (*n* = 4) [27]. The majority (13/19) of studies were conducted before the WHO PITC guidelines were developed in 2007, indicating that provider-initiated testing was occurring in many locations prior to the publication of global guidance. All of the studies that reported rates of HIV testing uptake showed increases in the HIV testing uptake associated with a PITC approach. The PITC’s impact on other outcomes does not appear to be worse than voluntary counselling and testing (VCT).

### Cost-Effectiveness

Three studies reported the cost-effectiveness of HIV testing strategies. Ekwueme [34] compared the costs of three HIV counselling and testing technologies, standard, one-step, and two-step rapid protocols. The standard protocol (i.e., ELISA) plus counselling and treatment, or one-step testing, was found to be less expensive than the third technology for all of the plausible ranges of HIV seroprevalence [34]. In low prevalence settings, a single rapid assay was cost-effective, as no follow-ups were required nor the use of the expensive Western blot confirmatory assay [34]. The second study, by Doyle [35], compared testing with an enzyme linked immunosorbent assay to rapid testing with Oraquick. In a low prevalence Mexican setting of 0.05%, rapid testing with Oraquick was the cost-effective strategy, at $217,718 per HIV case prevented. Assuming a 70-year lifespan, this equated to $3111 per life-year gained [35]. The third study, by Paltiel [36], compared testing with ELISA to the current practice (background testing OR presentation with opportunistic infections), in high (3%) prevalence, medium (1%) prevalence, and low (0.1%) prevalence settings. The addition of a one-off ELISA test was cost-effective compared to the current practice, for prevalence rates of 3% and 1%, but not cost-effective at a prevalence rate of 0.05% (incremental cost-effectiveness ratio: $113,000/QALY (Quality-adjusted Life Year) gained) [36].

The evidence supporting multiple rapid-tests, rather than a single rapid test followed by later confirmatory test if positive, were mixed. One study supported the use of a single rapid test [32], while another suggested possible cost savings with multiple rapid assays [34]. In this study, however, the cost advantage of multiple rapid assays was sensitive to HIV seroprevalence. In low prevalence settings (<0.1%), a single rapid assay was likely to be cost effective. The rapid tests evaluated in early economic studies were generally reported to have a lower sensitivity than ELISA tests [30,33]. Rapid testing is expanding to self-administered oral swabs. Of the limited economic evidence regarding rapid test false positives, one study [35] indicated a predictive value of 100% of the Oraquick rapid test, even in a low prevalence population (as low as 1 in 1000). Another study [36] assigned a loss of 14 quality-adjusted days to patients who received a false positive result from rapid testing.

## 4. Discussion

Our systematic review provides insight into HIV testing strategies to improve access and uptake in migrant populations in the EU/EEA, following the effectiveness and cost-effectiveness considerations.

**Figure 2:**
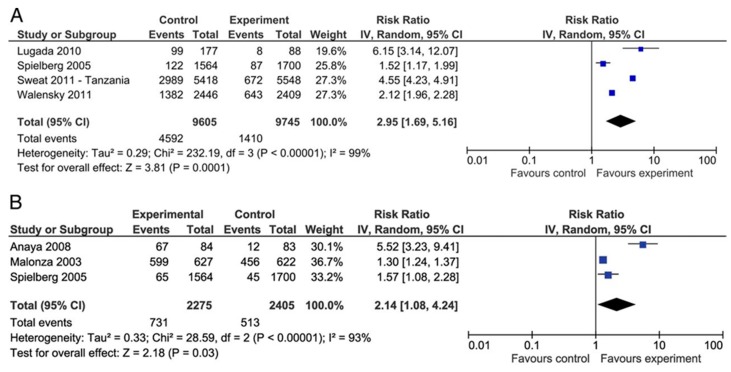
Meta-analysis (taken from Pottie et al., 2014 [26]). Forest plot of rapid HIV voluntary counselling and testing versus conventional care (**A**) on uptake of HIV testing and (**B**) on receipt of HIV results. Copyright received from BMJ. License Number 4403690523053.

In relation to our first research question, there were several HIV testing approaches. The literature showed three leading strategies, rapid testing, conventional testing, and universal testing approaches. Voluntary rapid tests improve HIV testing and uptake and have the potential to improve linkage to counselling and treatment for migrant populations. The universal opt-out testing approach has good intentions but lacks community outreach. Given the effectiveness of HIV treatment, measures and strategies are needed in order to increase the uptake of testing and to reduce the late diagnosis among migrant populations. However, heterogeneity between the results of the rapid testing approaches (I^2^ of 93–99% in Figure 2) and limited EU/EEA-specific data suggest inconsistency between studies, thereby limiting our confidence in the transferability of these results across the EU/EEA migrant population contexts. The cost-effectiveness of the intervention, however, suggests that rapid testing is preferable to conventional testing in several contexts, due, in particular, to effective testing and counselling integration.

There is no data on the cost-effectiveness and resource requirements of HIV testing in migrant populations in the EU/EEA. Indirect evidence from the United States and South Africa provide some insight into the resources required. We identified three studies on HIV testing strategies [34,35,36]. The economic evidence suggests that rapid testing is likely preferable to conventional testing across a range of contexts, largely due to the ability to more effectively integrate testing and counselling. One study supported the use of a single rapid test [32], while another suggested possible cost savings with multiple rapid assays [34]. The evidence supporting multiple rapid-tests, rather than a single rapid test followed by a later confirmatory test if positive, is mixed. In low prevalence settings (<0.1%), a single rapid assay may still be cost effective.

### 4.1. Implementation Issues

Identifying and addressing potential barriers to implementing effective and cost-effective HIV testing strategies can further promote access and uptake. Barriers to testing at organisational, provider (cultural competency), patient, and community levels in Europe include a perception of low risk, fear and stigma of the disease and disclosure, discrimination, financial limitations, poor access to care, lack of knowledge of where to obtain testing, and entitlement to medical care due to migration status [3,43,44]. The uncertain migration status among migrants to Europe is a barrier to preventive and health services in the EU/EEA [13,45]. This is especially true of undocumented migrants in the EU/EEA, as their uncertain legal status results in precarious living conditions, and discovery of their HIV status may risk deportation in certain countries [46]. HIV-related stigma is a significant barrier to HIV testing, in addition to challenges with socio-economic status, language barriers, and a poor understanding of European healthcare systems [44]. For many migrants, the barriers outweigh the advantages of testing and treatment [47], further perpetuating the HIV testing problem.

Migrant [13] populations in Sub-Saharan African [48] were more likely to be tested for HIV if they were of poor health, lost a child or sexual partner to HIV, ART was available, testing was a requirement for marriage preparation, enhanced confidentiality, had strong social networks and support [13,48], and had a history of sexually transmitted infections (STIs) [44]. Increasing cultural sensitivity and community engagement in counselling and in community-based approaches with outreach and mobilisation, were highlighted as ways to address and promote access and uptake [15], reflecting the WHO recommendations for vulnerable populations. More equity oriented research is needed to identify barriers to HIV testing in EU/EEA.

The ECDC antenatal screening for HIV, hepatitis B, syphilis, and rubella susceptibility highlights routine HIV testing for all pregnant women [49]. Certain high income countries such as the United States, Australia, Ireland, and Canada recommend testing refugees from HIV endemic areas within one month of arrival in primary health care settings. Such countries have national recommendations [50,51,52,53] for counselling and testing for refugee and migrant populations. Providing HIV testing during routine consultations was generally appreciated by users as an acceptable way to address user inhibitions in asking for it. Several Latin American migrants in Europe deemed compulsory testing as an acceptable public health measure, while healthcare practitioners reported feeling unprepared to communicate HIV positive results and adjust the flow of care [54]. Most migrants who reported knowing how to access HIV testing, preferred to receive information directly from community practitioners [55].

In general, HIV testing is cost-effective compared with no testing; the current focus is on which testing strategies are the most cost-effective in a migrant health context. The cost-effectiveness of rapid vs. conventional counselling and testing strengthens the need to increase access and uptake. The sensitivity analyses and analytic frameworks, however, were limited and demonstrate how this field is dynamic, as new rapid oral tests emerge. The cost-effectiveness data of rapid HIV testing in the EU/EEA were not available, but economic evidence about the integration of counselling, testing, and treatment was promising. The precise costs and benefits associated with rapid testing in a variety of EU/EEA member state contexts should be evaluated more closely in high-quality economic studies that directly compare various rapid testing assays to conventional testing with ELISA. Such research would need to provide quantitative evidence of the incremental cost-effectiveness of various strategies, including the uncertainty around these estimates.

### 4.2. Public Health Considerations

It is of particular importance to consider the challenges faced by undocumented migrants in order to increase the access and uptake of HIV testing and treatment programmes in the EU/EEA. We know from large clinical trials that treatment reduces onward transmission by 96% [56]. People living with undiagnosed HIV infection and those diagnosed with HIV but not yet on treatment contribute disproportionately to the number of new HIV infections [57]. Some of the contributing structural/organisational barriers to testing include a lack of migration status, lack of funding, availability of community based services, and discrimination in entitlement to care. More than half of the EU/EEA countries do not provide ART for undocumented migrants [12], further exacerbating the issue and reducing the likelihood that these individuals will come forward for testing. Certain EU/EEA countries have initiated public health screening programmes for migrants at high risk for HIV infection. The benefits of HIV testing in migrants, at both individual and community levels are recognized by many EU/EEA countries, but developing suitable and comprehensive migrant screening programmes has been a challenge in many countries [13].

### 4.3. Strengths and Limitations

One of the strengths of this review is that it has used GRADE methodology to assess the certainty of the evidence of the included studies, including recent systematic reviews from the U.S. AHRQ and the WHO guidelines, in combination with recent reviews on rapid testing. This review’s strengths lie in identifying barriers to accessing testing, and highlighting the cost-effectiveness of increasing the uptake of HIV testing for migrants. The barriers reported from Europe align with migrant HIV access barriers in other high-income countries.

We identified no migrant-specific HIV screening studies and therefore focused on studies that considered high HIV prevalence populations, many of which considered non EU/EEA migrants. This may limit the transferability of the findings to the EU/EEA context. We also acknowledge the lack of economic evidence for HIV testing approaches in migrants to the EU/EEA. The economic evidence is most relevant to the health system in which the study was undertaken, and therefore non-European studies may not be transferable. In addition, a few studies were more than twenty years old, and the costs may have changed since the resource use data was collected.

## 5. Conclusions

The migrants coming from countries with a high HIV prevalence often arrive with HIV related stigma and fear, and the screening and testing approaches need to address this challenge. HIV testing approaches that incorporate voluntary rapid testing programmes and primary care testing for high risk migrant populations may increase the uptake of testing, support timely diagnoses, and should provide more opportunities for linkage to effective treatment among migrant populations. All of the testing strategies may improve early diagnosis; treatment improves the individual’s clinical outcomes, reduces AIDS-defining events’ morbidity, and decreases mortality rates from HIV-related events, as well as having a clear public health benefit. Voluntary testing with rapid results offers an opportunity to overcome HIV related stigma in communities with high HIV prevalence compared to the conventional techniques for HIV testing alone.

## Figures and Tables

**Table 1:** Characteristics of included studies (effectiveness). HIV—human immunodeficiency virus; EU/EEA—European Union/European Economic Area; RCT—randomised-controlled trial; RR—relative risk; WHO—World Health Organization; CI—confidence interval; SMS—short message service.

Study	Design and Quality	Included Studies	Population	Intervention	Results/Outcomes
Should Voluntary Testing for HIV Infection be Offered to all Recently Arrived Migrants to the EU/EEA?					
Pottie et al., 2014 [26]	Systematic review AMSTAR 9/11	*n* = 13 Anaya et al. (RCT, *n* = 251, United States of America). Coates et al. (cRCT, *n* = 115,900, Tanzania, Zimbabwe, Thailand, and South Africa). Lugada et al. (cRCT, *n* = 7184, Uganda). Malonza et al. (RCT, *n* = 1249, Kenya). Read et al. (RCT, *n* = 400, Australia). Spielberg et al. (cRCT, *n* = 17,007, United States of America). Sweat et al. (cRCT, *n* = 57,156, Tanzania, Zimbabwe, and Thailand). Walensky et al. (RCT, *n* = 4855, United States of America). Appiah et al. (cross sectional, *n =* Not reported Ghana). Huebner et al. (Controlled before-after study *n* = NR, United States of America). Liang et al. (cohort, *n* = not reported United States of America). Shrestha et al. (cohort, *n* = not reported United States of America). White et al. (cohort, *n* = not reported, United States of America).	Individuals at high risk of exposure	Facilitated voluntary enrolment; use of a rapid-testing approach (providing results within 24 h); outreach counseling, delivery of results and treatment options.	Receipt of HIV test results: Increased likelihood among participants randomized to the rapid approach study arms to receive test results (RR = 2.14, 95% CI 1.08 to 4.24) (*n* = 3; RCTs). Repeat HIV testing and test incidence rate: increased HIV repeat testing among those in the intervention arm (RR = 2.28, 95% CI 0.35 to 15.07) (*n* = 1; cluster RCT). HIV incidence 36-month period in five countries showed an 11% reduction in estimated incidence in intervention RR = 0.89, 95% CI = 0.63 to 1.24). Treatment program uptake: OR = 1.7, 95% CI 0.8 to 3.7 for the uptake of perinatal HIV-1 interventions between rapid VCT versus conventional VCT (*n* = 1)
Kennedy et al., 2013 [27]	Systematic Review AMSTAR 5/11	*n* = 19 Allen et al. (time series, *n* = 1458, Rwanda). Allen et al. (non-randomized trial, *n* = not reported, Rwanda). Allen et al. (time series, *n* = 1438, Rwanda). Bentley et al. (time series, *n =* 1628, India). Brou et al. (time series, *n* = 980, Cote d’Ivoire). Chamdisarewa et al. (cross sectional, *n* = 4872, Zimbabwe). Creek et al. (cross sectional, *n* = 1456, Botswana). Desgrees-Du-Lou et al. (cohort, *n* = 937, Cote d’Ivoire). Harris et al. (cross sectional, *n* = not reported, Zambia). Huerga et al. (cross sectional, *n* = 409, Kenya). Khoshnood et al. (RCT, *n* = 600, China). Kiene et al. (before–after, *n* = 245, Uganda). Moses et al. (cross sectional, *n* = not reported, Malawi). Pang et al. (cross sectional, *n* = 585, China). Stringer et al. (cRCT, *n* = 246, Zambia). Van Rie et al. (nRCT, *n =* 1238, DRC). Van’t Hoog et al. (cross sectional, *n* = 4142, Kenya). Wiktor et al. (time series, *n* = 559, Cote d’Ivoire). Xu et al. (time series, *n* = 779, Thailand).	Low- and middle-income countries; health care setting where individuals were seeking health care services other than HIV testing. Individuals, couples, or groups had to receive pre- and post-test counseling about HIV and an HIV test	Provider-initiated testing and counseling (PITC) (aligned with the 2007 WHO).	The majority of studies were conducted before WHO PITC guidelines were developed, indicating that provider-initiated testing was occurring in many locations prior to global guidance. All studies included in this review that reported rates of HIV testing uptake showed increases associated with a PITC approach. Comparing behavior in the three months preceding PITC to behavior in the three months after PITC, the percentage of participants who reported engaging in risky sex decreased and knowing their partner’s HIV status increased for both HIV-positive and HIV-negative participants.
AHRQ 2012 [28]	Systematic Review AMSTAR 9/11	*n*= 42 Amaro et al. (before-after, *n* = 939, United States of America). Anglemyer et al. (systematic review, *n* = 8). Bedimo et al. (observational, *n* = 19,424, United States of America). Brogly et al. (before–after, *n* = not reported, Canada). Camoni et la (before–after, *n* = 487, Italy). Cohen et al. (RCT, *n* = 1763, Botswana, Kenya, Malawi, South Africa, Zimbabwe, India, Brazil, Thailand, and United States of America). Cunningham et al. (cross sectional, *n* = 300, United States of America). Data collection on Adverse events of Anti-HIV Drugs (DAD) study group (observational, *n* = 33,308, North America, Europe, and Australia). Das et al. (cohort, *n* = 12,512, United States of America). Del Romero et al. (cross sectional, *n* = 625, Spain). Donnell (pre-post, *n* = 3381, Botswana, Kenya, Rwanda, South Africa, Tanzania, Uganda, and Zambia). Diamond et al. (cross sectional, *n* = 886, United States of America). El-Bassel et al. (cRCT, *n* = 535, NR). Elford et al. (cross sectional, *n* = 1687, United Kingdom). Fideli et al. (case control, *n* = 109, Zambia). Fisher et al. (cohort, *n* = 859, United Kingdom). Fox et al. (before-after, *n* = 98, United Kingdom). Goncalyes Melo et al. (cohort, *n* = 93, Brazil). Haukoos et al. (cohort, *n* = not reported, United States of America). Hernando et al. (cohort, *n* = 399, Spain). HIV-CAUSAL (cohort, *n =* 62,760, 12 European cohorts). Kihata et al. (cohort, *n* = 17,517, North America). May et al.; Lanoy et al.; Moore et al. (cohort, *n* = 20,379, Europe and North America). Miguez-Burbbano et al. (cross sectional, *n* = 85, United States of America). Montaner et al. (cohort, *n* = 5413, Canada). Morin et al. (cross sectional, *n* = not reported, United States of America). Musicco (cohort, *n* = 436, Italy). Myers et al. (pre-post, *n* = not reported, United States of America). Obel et al., Lohse et al., 2006 (observational, *n* = 2952, Denmark). Reynolds et al. (cohort, *n* = 250, Uganda). Ribaudo et al. (observational, *n* = 5056). Severe et al. (RCT, *n* = 816, Haiti). SMART (RCT, *n* = 477, USA/Europe). Smit et al.; van Haastrecht et al. (cohort, *n* = 197, Amsterdam). Sullivan et al. (cohort, *n* = 2993, Rwanda and Zambia). Tun et al.; Vlahov et al. (before-after, *n* = 190, USA). Wang et al. (cohort, *n* = 1927, China). Weis et al. (corss sectional, *n* = not reported, United States of America). When to Start Consortium (cohort, *n* = 45,691, Europe and North America). White et al. (cohort, n-6479, United States of America). Wood et al. (cohort, *n* = 2051, Canada). Writing Committee for the CASCADE (Concerted Action on SeroConversion to AIDS and Death in Europe) Collaboration (cohort, *n* = 9455 Europe, Australia, and Canada).	Testing for asymptomatic HIV infection in Non-pregnant adults and adolescents.	Screening Strategies	No randomized trial or observational study compared clinical outcomes between adults and adolescents screened and not screened for HIV infection. Some modeling studies have estimated the cost-effectiveness of strategies involving repeat screening. No study directly evaluated the acceptability of universal versus targeted HIV screening. One study found universal, opt-out rapid screening associated with higher likelihood of testing compared with physician-directed, targeted rapid screening (25% vs. 0.8%; relative risk [RR], 30 [95% CI, 26 to 34]). One study found universal testing associated with a higher median CD4 count and lower likelihood of CD4 count <0.200 × 109 cells/L at the time of diagnosis compared with targeted HIV screening, but these differences were not statistically significant.
Desai et al., 2015 [29]	Systematic Review AMSTAR 6/11	*n* = 17 Bloomfeild et al. (observational, *n* = 399, United States of America). Bourne et al. (observational, *n* = 3551, Australia). Burton et al. (observational, *n* = 539, United Kingdom). Cameron et al. (observational, *n* = 330, United Kingdom). Cook et al. (RCT, *n* = 388, United States of America). Downing et al. (RCT, *n* = 94, Australia). Gotz et al. (RCT, *n* = 216, The Netherlands). Gotz et al. (observational, *n* = 4191, The Netherlands). Guy et al. (observational, *n* = 681, Australia). Harte et al. (observational, *n* = 301, United Kingdom). La Montagne (observational, *n* = 592, United Kingdom). Malotte et al. (RCT, *n* = 499, United States of America). Paneth-Pollak et al. (observational, *n* = 6220, United States of America). Sparks et al. (RCT, *n* = 122, United States of America). Walker et al. (observtional, *n* = 1116, Australia). Xu et al. (RCT, *n* = 1215, United States of America). Zou et al. (observational, *n* = 4179, Australia).	HIV-negative or unknown status in all countries; Hospitals, sexual health clinics, general practice, community venues, and home sampling/testing	Active recall	SMS: OR for retesting as compared to the control group ranged between 0.93 (95% CI 0.65 to 1.33) and 5.87 (95% CI 1.16 to 29.83). The pooled OR among the observational studies was 2.19 (95% CI 1.47 to 3.23). A pooled OR for retesting among SMS group is 5.66 (95% CI 1.78 to 17.99) among 126. Phone calls: phone calls and verbal advice and counseling had higher rates of retesting OR = 2.50 (95% CI 1.3 to 4.8) compared to phone calls only. Groups receiving phone calls and verbal advice had higher rates if retesting OR = 14.0 (95% CI 1.63 to 120.09) compared to phone calls only.

**Table 2:** Characteristics of included studies (cost-effectiveness). AIDS—acquired immunodeficiency syndrome.

Study	Quality/Drummond Score	Design	Population	Intervention	Cost Effectiveness	Resource Requirements
What are the Cost-Effectiveness and Resource Requirements of HIV Testing?						
Farnham et al., 1996 [30]	Allowance was made for uncertainty, sensitivity analysis performed around a variety of model inputs. One-way sensitivity analysis in a decision analytic framework. Sensitivity analysis compares basic value with the breakeven value that makes the two strategies equally cost-effective. No range of values tested and no a priori justification for values tested in sensitivity analysis. There was no assumed range, as noted above, but results seem to be sensitive to plausible changes in some model inputs, especially waiting and counselling times.	Decision analytic model, societal perspective. Costs measured in 1992 U.S. dollars.	United States of America	ELISA test, and counselling and testing (C/T) vs. rapid C/T vs. no intervention	ELISA C/T: Average not incremental cost-effectiveness ratios: $1165 per correctly identified case vs. no intervention; rapid C/T $940 per correctly identified case Rapid vs. ELISA: $596 per correctly identified case	ELISA C/T: positive individual $103 per person, negative individual $33. Rapid C/T: positive individual $135 per person, negative individual $33 per person. Low to moderate costs of both strategies.
Kassler et al., 1997 [31]	No allowance was made of uncertainty. No sensitivity analyses.	Cost comparison, societal perspective. Comparison of testing strategies in an HIV clinic. Costs measured in 1993 U.S. dollars.	Individuals attending an anonymous testing clinic and a sexually transmitted disease (STD) clinic in Dallas, Texas	Standard C/T vs. rapid C/T	No incremental cost-effectiveness ratio calculated, not a full economic evaluation.	Cost per person receiving results and counselling: standard $151, rapid $131. Low to moderate cost savings of rapid C/T over standard C/T.
Wilkinson et al., 1997 [32]	No allowance was made for uncertainty. No sensitivity analyses.	Cost comparison, prospective comparison of testing strategies in a South African hospital. Costs measured in 1996 South African rand.	Resource-poor setting: adult inpatients of a rural South African district hospital	ELISA C/T vs. single rapid C/T vs. double rapid C/T. The double rapid strategy consists of two different rapid tests: a Capillus test and an Abbott test.	N/A	Cost per person counselled post-test: single rapid R 14–31.2, double rapid R 45.2, ELISA R 83.8. Cost savings of single rapid test.
Kallenborn et al., 2001 [33]	Some allowance made for uncertainty. Sensitivity analysis limited and discursive. Not statistically rigorous. No range of values for sensitivity analysis provided. Results overall not sensitive to whether basic or expanded regimen used, but as noted sensitivity analysis was incomplete.	Cost comparison study, retrospective chart review of Health Care Workers in an emergency department. Costs measured in 1999 U.S. dollars.	Healthcare workers	Rapid testing vs. ELISA testing	N/A. This is just a cost comparison	Total costs for 17 patients: ELISA $5966, Rapid test $466. Cost savings of switching from ELISA testing to rapid testing in health care workers.
Ekwueme et al., 2003 [34]	Allowance made for uncertainty. One-way sensitivity analysis performed in a cost analysis model. Range of sensitivity analysis is +/− 50% of the base value, or as wide as possible in the absence of hard data. Rank order of two-step rapid relative to standard C/T sensitive to the return rate for standard C/T, but one-step rapid consistently least expensive.	Cost analysis study using a decision analysis model, costs estimated from both societal and provider perspective, in 2000 U.S. dollars	United States of America	Standard ELISA C/T vs. both one-step (multiple rapid assays) and two-step rapid C/T (i.e., with a confirmatory Western blot test)	N/A	From both a provider and societal perspective, costs vary based on sero-status. However, one-step rapid testing is consistently the lowest cost option, and two-step rapid testing tends to be the highest cost. There appear to be cost savings of using a one-step rapid C/T protocol vs. standard ELISA testing or two-step rapid C/T.
Doyle et al., 2005 [35]	Allowance made for uncertainty. One-way sensitivity analysis on “sensitivity, specificity, and positive predictive values of each screening test and confirmatory Western blot test, the costs of each test, and the costs of treatments” in a decision analytic model. Range of values tested in sensitivity analysis appears to be based on published estimates but this is not explicitly stated. The Oraquick rapid test is the dominant strategy over a wide range of assumptions. Results not sensitive to plausible changes.	Decision analysis techniques: decision tree	Low risk Mexican American population, incidence 0.05%	(1) testing with enzyme linked immunosorbent assay that was confirmed by Western blot (2) testing with Oraquick rapid testing that was confirmed by Western blot	Oraquick as the primary screening test for the unknown HIV status of women who were in labor was the most cost-effective at $217,718 per HIV case that was prevented. Assuming a 70-year lifespan, this equals $3111 per life-year gained.	Oraquick cost $98 spent for each child who was HIV negative, ELISA screening cost $491. High cost of ELISA screening in a low-prevalence Mexican American population were from unnecessary treatment of women and infants with false-positive test results. Oraquick has a relatively modest costs.
Paltiel et al., 2005 [36]	Allowance made for uncertainty One-way sensitivity analysis performed. Range of estimates seems to be derived from published estimates/a plausible a priori estimate. Not explicitly stated. Some sensitivity to assumptions regarding background therapy, adherence to ARV therapy, and rates of linkage to care. This does not significantly change the results, may simply change whether screening every three or five years is preferable. Results regarding rapid vs. conventional testing are unclear and sensitive to plausible changes in background testing rates, acceptance and linkage to care, and rate of secondary transmission.	A stochastic model (individual model) of HIV screening and treatment: The cost-effectiveness of preventing AIDS, and a complications model (CEPAC model)	United States of America	(i) routine, voluntary HIV, CTR (counselling, testing and referral); (ii) current practice: background testing OR presentation with opportunistic infections in three target populations:	Compared to current practice, current practice plus one time ELISA costs $36,000 per Quality-Adjusted Life Year (QALY) gained; current practice plus ELISA every 5 years costs $50,000 per QALY gained; current practice plus ELISA every 3 years costs $63,000; current practics plus ELISA every year costs $100,000 per QALY gained	For HIV infected persons only: current practice costs: $78,100 lifetime cost per person; current practice plus one time ELISA costs $80,700; current practice plus ELISA every five years costs $89,000; current practice plus ELISA every 3 years costs $92,500; current practices plus ELISA every year costs $98,600 For general population: current practice costs $32,700 lifetime cost per person, current practice plus one-time ELISA costs $33,800; current practice plus ELISA every five years costs $37,300 current practice plus ELISA every three years costs $38,900; current practices plus ELISA every year costs $41,700. More frequent screening produced large costs, due to screening test cost plus the cost of managing false positives.
Vickerman et al., 2006 [37]	Allowance made for uncertainty. Univariate sensitivity analysis for a number of dimensions. Model input ranges derived from published estimates. The cost-effectiveness of the POC rapid test is sensitive to test cost.	Dynamic compartmental model	Female sex workers	A range of sensitivities of point of care (POC) tests.	If the POC test cost $2 per test (2004 $US), and was 70% sensitive, then POC test would cost $152 per additional HIV infection averted, which is cost-effective. If the cost of the POC test was $1 and the sensitivity was 80%, the cost per HIV infection averted would have been $58, which is cost-effective. When the POC test has a low sensitivity, of 50%, POC is not cost-effective.	Possible cost savings from using POC tests include the reduction in the number of STI clinic attenders receiving treatment. Assuming each test takes an extra 0.3 h to undertake, POC testing costs for 4 years is $13,399 if the test cost $1 and $34 621 if the test cost $3 (in 2004 U.S.$). Moderate costs.

**Table 3:** Recommendations Assessment, Development, and Evaluation (GRADE) evidence profile.

Certainty Assessment							Effect				Certainty	Importance
No. of Studies	Study Design	Risk of Bias	Inconsistency	Indirectness	Imprecision	Other Considerations	Relative (95% CI)	Absolute (95% CI)				
Outcome: Testing Uptake (follow up: 7 to 24 months)												
3 (9745 participants)	Randomised trials	Serious concerns, allocation concealment was unclear, blinding of intervention not possible, and inability to determine blinding of researchers	No serious inconsistency	No serious indirectness	No serious imprecision	No other concerns	RR 2.95 (1.69 to 5.16)	Without rapid testing for HIV	With rapid testing for HIV	Difference	Moderate	Critical
								General population				
								0.1%	0.3% (0.2 to 0.5)	0.2% more (0.1 more to 0.4 more)		
								High risk population				
								2.0%	5.9% (3.4 to 10.3)	3.9% more (1.4 more to 8.3 more)		
Outcome: HIV incidence (follow up: 36 months)												
1 (8324 participants)	Randomised trial	Serious concerns Allocation concealment was unclear, blinding of intervention not possible and inability to determine blinding of researchers	No serious inconsistency	No serious indirectness	Serious imprecision	No other concerns	RR 0.89 (0.63 to 1.24)	Low risk population			Low	Critical
								7.2%	6.4% (4.5 to 8.9)	0.8% fewer (2.7 fewer to 1.7 more)		

The risk in the intervention group (and its 95% confidence interval) is based on the assumed risk in the comparison group and the relative effect of the intervention (and its 95% CI). CI—confidence interval; RR—risk ratio; GRADE working group grades of evidence: high quality: we are very confident that the true effect lies close to that of the estimate of the effect; moderate quality: we are moderately confident in the effect estimate, the true effect is likely to be close to the estimate of the effect, but there is a possibility that it is substantially different; low quality: our confidence in the effect estimate is limited: The true effect may be substantially different from the estimate of the effect; very low quality: we have very little confidence in the effect estimate, the true effect is likely to be substantially different from the estimate of effect. Interpreting relative values (e.g., uptake of testing) from the summary of findings table: relative risk: (RR 2.98; 95% CI: 1.69–5.16)-three RCTs included in the analysis provided consistent point estimates showing that the uptake of testing was 2.95 times better among participants randomized to rapid testing approaches.

**Figure A1:**
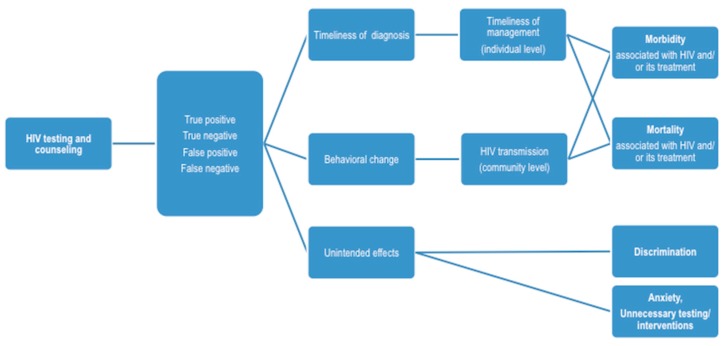
Logic Model.

## Appendix B. Search Strategy

Database: Ovid MEDLINE(R) 1946 to Present with Daily Update

Search Date: 8 April 2016

1. (hiv or hiv1$ or hiv2$).mp. (277,362)
2. (human adj (immunedeficienc$ or immune deficienc$ or immunodeficienc$ or immuno deficienc$)).tw. (72,052)
3. Acquired Immunodeficiency Syndrome/(74,163)
4. (acquired adj (immunedeficienc$ or immune deficienc$ or immunodeficienc$ or immuno deficienc$)).tw. (20,316)
5. aids.hw. (59,809)
6. (aids adj2 (infect$ or virus$)).tw. (5953)
7. or/1–6 (346,871)
8. exp Mass Screening/(107,701)
9. (screened or screening?).tw. (417,187)
10. Early Diagnosis/(18,989)
11. [or/8–15] (0)
12. [or/17–19] (0)
13. [remove duplicates from 24] (0)
14. (hiv or hiv1$ or hiv2$).mp. (277,362)
15. (human adj (immunedeficienc$ or immune deficienc$ or immunodeficienc$ or immuno deficienc$)).tw. (72,052)
16. Acquired Immunodeficiency Syndrome/(74,163)
17. (acquired adj (immunedeficienc$ or immune deficienc$ or immunodeficienc$ or immuno deficienc$)).tw. (20,316)
18. aids.hw. (59,809)
19. (aids adj2 (infect$ or virus$)).tw. (5953)
20. or/14–19 (346,871)
21. exp Mass Screening/(107,701)
22. (screened or screening?).tw. (417,187)
23. Early Diagnosis/(18,989)
24. ((case? or early) adj2 (detected or detection? or diagnos$ or discover$)).tw. (149,145)
25. exp Population Surveillance/(55,996)
26. (disease? adj2 surveillance).tw. (4047)
27. Contact Tracing/(3517)
28. contact tracing.tw. (1151)
29. or/21–28 (646,541)
30. meta analysis.mp,pt. (90,987)
31. review.pt. (2,033,544)
32. search$.tw. (253,118)
33. or/30–32 (2,219,856)
34. animals/not (humans/and animals/) (4,191,261)
35. 33 not 34 (2,063,224)
36. 20 and 29 and 35 (3419)
37. 36 and (2010$ or 2011$ or 2012$ or 2013$ or 2014$ or 2015$ or 2016$).ed. (1178)
38. remove duplicates from 37 (1137)

***************************

Database: Embase <1980 to 7 April 2016 >

Search Date: 8 April 2016

1. (hiv or hiv1$ or hiv2$).mp. (313,646)
2. exp Human immunodeficiency virus infection/(322,600)
3. exp Human immunodeficiency virus/(156,358)
4. (human adj (immunedeficienc$ or immune deficienc$ or immunodeficienc$ or immuno deficienc$)).tw. (81,168)
5. (acquired adj (immunedeficienc$ or immune deficienc$ or immunodeficienc$ or immuno deficienc$)).tw. (22,202)
6. aids.hw. (11,098)
7. (aids adj2 (infect$ or virus$)).tw. (6778)
8. or/1–7 (447,157)
9. exp mass screening/(178,092)
10. (screened or screening?).tw. (612,553)
11. anonymous testing/(221)
12. early diagnosis/(82,014)
13. ((case? or early) adj2 (detected or detection? or diagnos$ or discover$)).tw. (223,587)
14. exp health survey/(182,039)
15. (disease? adj2 surveillance).tw. (5133)
16. contact examination/(2820)
17. contact tracing.tw. (1443)
18. or/9–17 (1,076,977)
19. meta analys$.mp. (166,352)
20. search$.tw. (360,207)
21. review.pt. (2,126,810)
22. or/19–21 (2,466,318)
23. animals/not (humans/and animals/) (1,150,973)
24. 22 not 23 (2,401,775)
25. 8 and 18 and 24 (5168)
26. 25 and (2010$ or 2011$ or 2012$ or 2013$ or 2014$ or 2015$ or 2016$).dd. (2136)
27. remove duplicates from 26 (2080)

***************************

Databases: Database of Abstracts of Reviews of Effects (DARE) and Cochrane Database of Systematic Reviews (CDSR)

Search Date: 20 March 2016

ID Search
#1 (hiv or hiv1* or hiv2*) (15,056)
#2 human next (immunedeficienc* or immune deficienc* or immunodeficienc* or immuno deficienc*):ti,ab (2799)
#3 MeSH descriptor: [Acquired Immunodeficiency Syndrome] this term only (1248)
#4 acquired next (immunedeficienc* or immune deficienc* or immunodeficienc* or immuno deficienc*):ti,ab (646)
#5 aids:kw (2253)
#6 aids near/2 (infect* or virus*):ti,ab (457)
#7 #1 or #2 or #3 or #4 or #5 or #6 (16,330)
#8 MeSH descriptor: [Mass Screening] explode all trees (5443)
#9 (screened or screening*):ti,ab (22,416)
#10 MeSH descriptor: [Early Diagnosis] this term only (538)
#11 (case* or early) near/2 (detected or detection* or diagnos* or discover*):ti,ab (3639)
#12 MeSH descriptor: [Population Surveillance] explode all trees (709)
#13 disease* near/2 surveillance:ti,ab (30)
#14 MeSH descriptor: [Contact Tracing] this term only (96)
#15 contact tracing:ti,ab (30)
#16 #8 or #9 or #10 or #11 or #12 or #13 or #14 or #15 (27,248)
#17 #7 and #16 (1173)
#18 #17 in Other Reviews (27)
#19 #17 in Cochrane Reviews (Reviews and Protocols) (195)

***************************

Database: EBSCO CINAHL <1970 to April 2016>

Search Date: 8 April 2016

S27 S23 AND S26 297
S26 S24 OR S25 2,588,490
S25 EM 2010 or EM 2011 or EM 2012 or EM 2013 or EM 2014 or EM 2015 or EM 2016 2,411,599
S24 PY 2010 or PY 2011 or PY 2012 or PY 2013 or PY 2014 or PY 2015 or PY 2016 2,338,383
S23 S8 AND S16 AND S22 527
S22 S17 OR S18 OR S19 OR S20 OR S21 220,800
S21 (TI meta analy* or AB meta analy*) 29,599
S20 (MH “Meta Analysis”) 24,899
S19 PT review 141,121
S18 PT systematic review 53,358
S17 (MH “Systematic Review”) 37,370
S16 S9 OR S10 OR S11 OR S12 OR S13 OR S14 OR S15 154,560
S15 contact tracing 1457
S14 (disease* or population) N2 surveillance 18,647
S13 (MH “Population Surveillance+”) 5939
S12 (case* or early) N2 (detected or detection* or diagnos* or discover*) 29,738
S11 (MH “Early Diagnosis”) 4469
S10 TI ((screened or screening*)) OR AB ((screened or screening*)) 78,064
S9 (MH “Health Screening+”) 62,689
S8 S1 OR S2 OR S3 OR S4 OR S5 S6 OR S7 108,656
S7 TX aids N2 (infect* or virus*) 10,961
S6 MW aids 22,515
S5 TX acquired N1 (immunedeficienc* or immune deficienc* or immunodeficienc* or immuno deficienc*) 21,283
S4 (MH “Acquired Immunodeficiency Syndrome”) 13,583
S3 TX human N1 (immunedeficienc* or immune deficienc* or immunodeficienc* or immuno deficienc*) 23,570
S2 (hiv or hiv1* or hiv2*) 79,932
S1 (MH “Human Immunodeficiency Virus+”) 6511

## Appendix C

**Table A1:** Population, intervention, comparison, and outcome (PICO) inclusion criteria.

**Population:**	Migrants and refugees to EU/EEA countries (primary population of interest); will consider indirect evidence of marginalized groups in settings of high HIV prevalence
**Intervention:**	Voluntary testing for HIV
**Outcome:**	Testing outcomes: testing uptake, HIV incidence Treatment outcomes: Efficacy, withdrawals
